# Homer regulates calcium signalling in growth cone turning

**DOI:** 10.1186/1749-8104-4-29

**Published:** 2009-08-03

**Authors:** Robert Gasperini, Derek Choi-Lundberg, Michael JW Thompson, Camilla B Mitchell, Lisa Foa

**Affiliations:** 1 School of Medicine, University of Tasmania, Hobart, 7001, Tasmania, Australia; 2 Menzies Research Institute, University of Tasmania, Hobart, 7001, Tasmania, Australia

## Abstract

**Background:**

Homer proteins are post-synaptic density proteins with known functions in receptor trafficking and calcium homeostasis. While they are key mediators of synaptic plasticity, they are also known to function in axon guidance, albeit by mechanisms that are yet to be elucidated. Homer proteins couple extracellular receptors – such as metabotropic glutamate receptors and the transient receptor potential canonical family of cation channels – to intracellular receptors such as inositol triphosphate and ryanodine receptors on intracellular calcium stores and, therefore, are well placed to regulate calcium dynamics within the neural growth cone. Here we used growth cones from dorsal root ganglia, a well established model in the field of axon guidance, and a growth cone turning assay to examine Homer1 function in axon guidance.

**Results:**

Homer1 knockdown reversed growth cone turning from attraction to repulsion in response to the calcium-dependent guidance cues brain derived neurotrophic factor and netrin-1. Conversely, Homer1 knockdown had no effect on repulsion to the calcium-independent guidance cue Semaphorin-3A. This reversal of attractive turning suggested a requirement for Homer1 in a molecular switch. Pharmacological experiments confirmed that the operational state of a calcium-calmodulin dependent protein kinase II/calcineurin phosphatase molecular switch was dependent on Homer1 expression. Calcium imaging of motile growth cones revealed that Homer1 is required for guidance-cue-induced rise of cytosolic calcium and the attenuation of spontaneous cytosolic calcium transients. Homer1 knockdown-induced calcium transients and turning were inhibited by antagonists of store-operated channels. In addition, immunocytochemistry revealed the close association of Homer1 with the store-operated proteins TRPC1 and STIM1 within dorsal root ganglia growth cones.

**Conclusion:**

These experiments provide evidence that Homer1 is an essential component of the calcium signalling repertoire within motile growth cones, regulating guidance-cue-induced calcium release and maintaining basal cytosolic calcium.

## Background

Deciphering the cell signalling events that control growth cone navigation and, hence, axon guidance is crucial to our understanding of the development of functional neural circuitry. Cytosolic calcium ([Ca^++^]_i_) is a key signalling molecule that regulates growth cone motility [1,2]. The release of calcium from intracellular stores or influx via receptor-mediated or voltage-gated channels leads to discrete localised transients and/or global changes in [Ca^++^]_i _[3]. The frequency and magnitude of these [Ca^++^]_i _changes correlates with overall axon growth and extension as well as responses to soluble and contact-mediated guidance cues [2,4-6]. These changes in [Ca^++^]_i _can be of the order of milliseconds or quite persistent, lasting several minutes [6,7]. Changes in spatial growth cone [Ca^++^]_i _gradients mediate the activation of calcium-calmodulin dependent protein kinase II (CaMKII) and calcineurin phosphatase (CaN) in a molecular switch-like mechanism that controls calcium-dependent growth cone turning [8]. This molecular switch is consistent with the [Ca^++^]_i _set point hypothesis [1,9,10] that predicts baseline [Ca^++^]_i _and/or frequency of transients is maintained at a low level, in order for discrete and/or global changes in [Ca^++^]_i _to be instructional to growth cone extension [11]. Calcium is a promiscuous second messenger and the complete molecular repertoire that regulates basal [Ca^++^]_i _and guidance-cue-induced changes in [Ca^++^]_i _within growth cones is yet to be fully resolved.

Homer proteins are cytosolic scaffold proteins that facilitate signalling at the dendritic post-synaptic density [12]. However, expression of Homer proteins is not restricted to the post-synaptic density, but rather they are expressed throughout neurons, including growth cones [13,14]. Consistent with an axonal growth cone distribution, Homer1b/c is known to be necessary for axon guidance *in vivo*. In the *Xenopus *visual system, a critical level of Homer1b/c, or long-form Homer1, is required for appropriate axon guidance and target recognition, where it acts cell-autonomously, presumably within the growth cone [14]. There are three separate Homer genes, all with multiple isoforms [15,16]. Homer1 has been studied extensively for its role in calcium signalling [17]. Long-form Homer, including Homer1b/c, forms homo- and hetero-tetramers with other Homer proteins via a carboxy-terminal coiled-coil domain and cross-links multiple signalling partners through an amino-terminal, enabled-VASP homology (EVH1) domain [16,18]. This structure enables Homer proteins to bind cell surface receptors such as metabotropic glutamate receptors and transient receptor potential canonical (TRPC) channels and couple them to intracellular calcium stores via the inositol triphosphate (IP_3_) receptor (IP_3_R) and ryanodine receptors [12,15,19,20]. Such scaffolding functions are known to enhance molecular signalling in many systems [21], yet it is not known how Homer facilitates molecular signalling during axon guidance.

Spatiotemporal patterns of calcium are believed to underpin growth cone motility and, through EVH1-binding partners, Homer would be predicted to be a regulator of calcium signalling within the growth cone [19]. Those binding partners include IP_3 _and ryanodine receptors on intracellular calcium stores and cation permeable TRPC channels on the plasma membrane [19,20]. In non-neuronal cells, Homer has been shown to couple IP_3_R to TRPC channels, thereby gating calcium influx and store release of intracellular calcium [20]. In neuronal cells, such a role would suggest that Homer may regulate calcium-induced calcium release (CICR). Longer, sustained global signals [3] are thought to be due to CICR, triggered by extracellular calcium influx and/or guidance cue activation of G-protein coupled receptors, in turn activating store release of calcium via IP_3 _or ryanodine receptors in the endoplasmic reticulum (ER). CICR causes a moderate rise in calcium and is required for growth cone attraction towards guidance cues, activating transport of membrane components to the leading edge of the growth cone [6,22-25]. Calcium influx through TRPC channels is also triggered after store depletion by the ER calcium sensing molecule stromal interacting molecule (STIM)1 [26]. STIM1 and STIM2 were recently described as calcium sensing proteins and have been demonstrated to be exquisitely sensitive to the concentration of calcium in their immediate environment [27]. While STIM2 appears to be primarily a sensor for cytosolic calcium, STIM1 is a sensor for calcium within the ER [28,29]. Upon ER depletion, STIM1 translocates as puncta within the ER membrane to regions closely associated with the plasma membrane, where it binds Orai and TRPC proteins to effect refilling of depleted calcium stores [26,30-32]. Interestingly, STIM1 possesses the Homer binding motif PXXF [33]. The presence of this binding site suggests the potential for Homer to mediate TRPC-STIM1 interactions [26]. Whether IP_3_R-Homer-TRPC protein or putative STIM1-Homer-TRPC protein interactions are important in neurons is not known. TRPC channels, however, are required for growth cone attraction to brain derived neurotrophic factor (BDNF) and Netrin-1 [34,35]. Furthermore, refilling of depleted stores, a process termed capacative calcium entry, or store-operated calcium entry [36], is likely to be crucial in growth cone motility. Therefore, the association of Homer with key calcium storage and regulatory partners makes it a potentially important molecule in the facilitation of calcium signalling within the growth cone.

In these experiments we demonstrate that Homer1 is a crucial regulator of calcium-dependent growth cone turning. We show that Homer1 knockdown reversed growth cone responses to calcium-dependent guidance cues from attraction to repulsion. Our data suggest that Homer1 regulates the operational state of a CaMKII-CaN molecular switch. Furthermore, Homer1 is required for guidance-cue-induced rises in [Ca^++^]_i _and attenuating the frequency of spontaneous calcium transients in motile growth cones. These data implicate Homer1 in mediating calcium influx via store-operated channels, with direct consequences for CICR, store-operated calcium entry and regulation of basal cytosolic calcium, all necessary for accurate directional control of growth cone motility.

## Results

### Homer1 expression is crucial for growth cone turning

**Figure 1:**
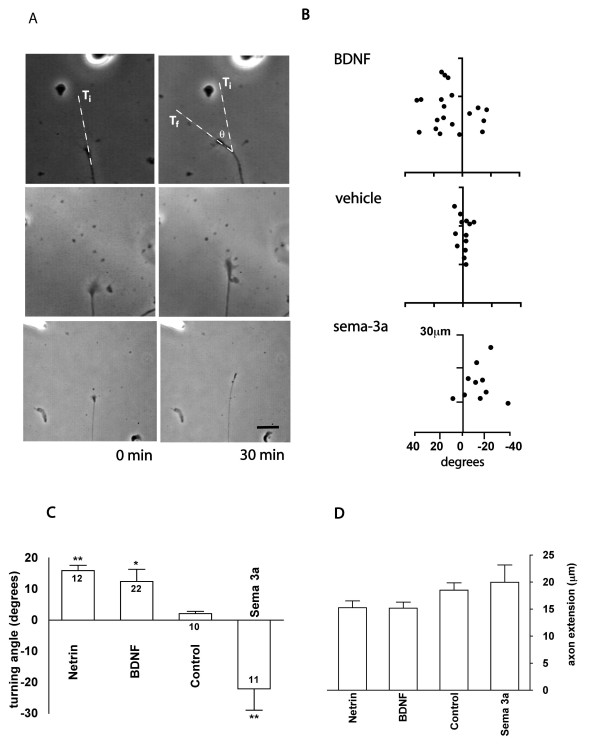
**Dorsal root ganglia (DRG) growth cone responses to brain derived neurotrophic factor (BDNF), Netrin-1 and Sema-3a in an *in vitro *turning assay**. **(A) **Representative time-lapse images of DRG growth cones at start (0 minutes) and end (30 minutes). T_i _= initial trajectory; T_f _= final trajectory; θ = turning angle. Scale bar is 10 μm. **(B) **growth cone extension/trajectory plots after a 30-minute exposure to gradients of BDNF, vehicle (sensory neuron medium) and Sema-3a. In all cases the micropipette is positioned out of image at upper left quadrant. Quantification of average turning angles **(C) **and axon extension rates **(D)**. Axon extension rates did not differ significantly after 30 minutes for Netrin-1, BDNF, control or Sema-3a. Positive angles represent attraction; negative angles represent repulsion. Significant differences from control values are marked as: *P < 0.05; **P < 0.005; Mann-Whitney *U*-test. Error bars indicate standard error of the mean.

Dorsal root ganglia (DRG) sensory neurons are a well-established model for axon guidance and growth cone motility studies [37]. We characterised the behaviour of embryonic rat DRG growth cones in an *in vitro *growth cone turning assay [38]. Using experimental parameters comparable to those used in other cell types [34,35], DRG growth cones showed reliable responses to attractive and repulsive guidance cues (Figure 1). Isolated DRG growth cones in acute primary culture turned towards micro-gradients of BDNF and Netrin-1, and were repelled by Semaphorin-3a (Sema-3a) when compared to vehicle-only experiments (Figure 1A–C). These effects were specific to turning and did not affect other cytoskeletal events, since axon extensions did not differ significantly between guidance cues (Figure 1D).

**Figure 2:**
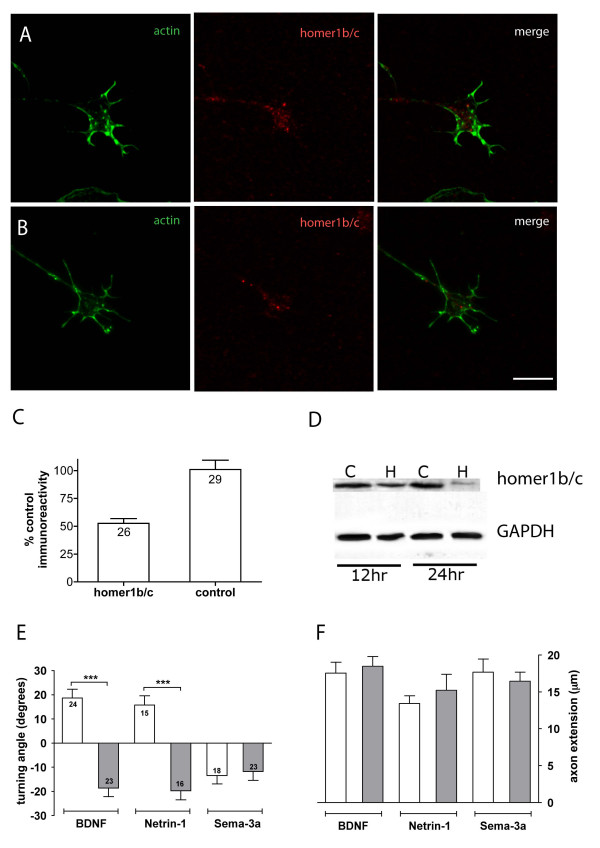
**Specific anti-sense morpholino knockdown of Homer1 expression causes a reversal in growth cone turning**. **(A) **Control morphant and **(B) **Homer1 morphant growth cones labelled for Homer1b/c (red) and f-actin (green) after 6 hours in culture. **(C) **Homer1 knockdown in growth cones was quantified by determining pixel intensity corrected for growth cone area and normalised to control morphant growth cones (100%). Numbers indicate growth cones imaged over three separate dishes. **(D) **Western blot of B-35 neuroblastoma cell protein extracts loaded with control (C), and Homer1 (H) morpholinos. Partial knockdown was achieved at 12 hours and significant knockdown at 24 hours. **(E) **Average growth cone turning angles after 30 minutes in gradients of Netrin-1, brain derived neurotrophic factor (BDNF) or Sema-3a after a 4 to 6 hour treatment with control morpholino (open bars) or Homer1 morpholino (shaded bars). **(F) **Control or Homer1 morpholinos did not have any significant effect on overall axon extension rates (compare Figures 1B and 2E). Significant differences from control values are marked as: ***P < 0.0005; Mann-Whitney *U*-test. Error bars indicate standard error of the mean. Scale bar for (A, B) is 5 μm.

Previously, over-expression experiments demonstrated the requirement of Homer1b/c in axon guidance *in vivo*, although the exact molecular mechanism has not been determined [14]. In the current study, we used a targeted anti-sense morpholino oligonucleotide knockdown approach to examine the role of endogenous Homer1 in the regulation of growth cone motility. Morpholinos have been used extensively in vertebrate model systems to effectively knockdown expression of proteins without the off-target effects seen with RNA interference [39]. In growth cones loaded with a control mis-primed Homer1 morpholino (control morphants), robust expression of Homer1b/c protein was apparent throughout the growth cone (Figure 2A). Treatment with a specific Homer1 morpholino (Homer morphants) significantly reduced Homer1b/c expression in isolated DRG growth cones after 6 hours *in vitro *(Figure 2B, C). Specific Homer1 knockdown was confirmed by western blot analysis of extracts from a human neuroblastoma cell line (B-35) treated with the same morpholinos for 12 or 24 hours (Figure 2D). Treatment with control or Homer1-specific morpholinos did not appreciably alter growth cone morphology (for example, compare Figures 2A and 2B). In embryonic DRG tissue, very little to no endogenous Homer1a protein is detectable by western blot analysis (data not shown), so the effects we report here are likely due to knockdown of endogenous Homer1b/c and not the activity induced isoform, Homer1a.

To examine the role of Homer1 in growth cone turning, we asked whether Homer1 knockdown would perturb growth cone responses in a turning assay. Homer1 morphants exhibited a dramatic reversal of attraction to repulsion in response to BDNF and Netrin-1. Conversely, treatment with the Homer1 morpholino had no effect on growth cone turning in response to Sema-3a (Figure 2E). The guidance cues BDNF and Netrin-1 are known to differ from Sema-3a in their downstream signalling effectors: BDNF and Netrin-1 require calcium signalling, while Sema-3a signalling is calcium independent [40]. Treatment with control morpholino had no affect on turning responses to BDNF, Netrin-1 or Sema3a (compare Figures 1C and 2E). Overall axon extension was not significantly different in control or Homer1 morphants compared to untreated growth cones (compare Figures 1D and 2F), confirming that morpholino treatment did not interfere with cytoskeletal rearrangements necessary for axon growth. Since Homer1 knockdown reversed turning only in response to calcium-dependent guidance cues, the data strongly suggests a necessary role for Homer1 in calcium signalling within the growth cone.

### Homer1 knockdown alters the operational state of the CaMKII-CaN molecular switch

**Figure 3:**
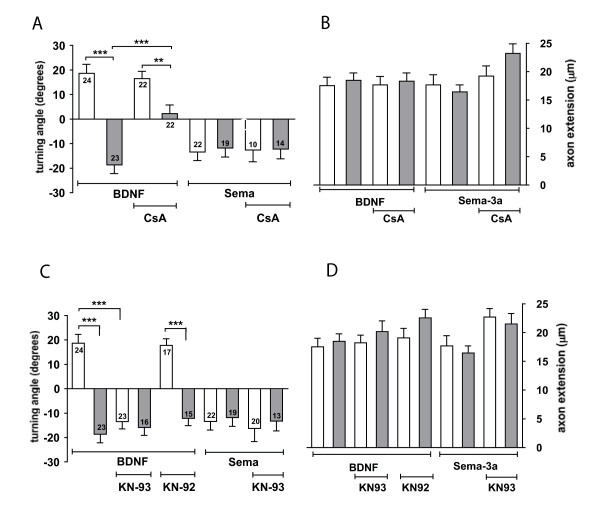
**Homer signalling operates in a calcium-dependent manner and regulates the operational state of a calcium-calmodulin dependent protein kinase II-calcineurin phosphatase molecular switch**. **(A, C) **Average growth cone turning angles for control (open bars) and Homer (shaded bars) morphant growth cones in response to brain derived neurotrophic factor (BDNF) and Sema-3a gradients following a 20 minute bath application of (A) cyclosporinA (CsA) or **(C) **KN93 and KN92. **(B, D) **Axon extension rates were not significantly different amongst control and Homer1 morphants with the same pharmacological treatments. Significant differences from control values are marked as: **P < 0.005; ***P < 0.0005; Mann-Whitney *U*-test. Error bars indicate standard error of the mean.

In motile growth cones, calcium transients and gradients underpin specific directional responses to guidance cues [7]. Experiments utilising focal uncaging of photo-activatable [Ca^++^]_i _have implicated a CaMKII-CaN molecular switch as a mechanism for the transduction of local and global calcium gradients into either attractive or repulsive responses to guidance cues. Relatively large changes in [Ca^++^]_i _activate CaMKII to induce attraction, while small changes in [Ca^++^]_i _activate CaN to effect repulsion [8]. Since Homer1 knockdown resulted in a reversal of growth cone responses from attraction to repulsion to the calcium-dependent cues BDNF and Netrin (Figure 2E), we asked whether Homer1 might function through such a molecular switch. We combined bath application of cyclosporin A (10 nM), an inhibitor of CaN, or KN-93 (5 μM), a specific inhibitor of CaMKI, II and IV, with morpholino treatment in the growth cone turning assay. The addition of cyclosporin A to Homer1 morphants abolished growth cone repulsion to BDNF, resulting in random turning (Figure 3A). Control morphant responses to BDNF were not affected by cyclosporin A treatment (Figure 3A), consistent with attraction being dependant on CaMKII activation [8]. Cyclosporin A had no effect on Sema-3a turning irrespective of Homer1 expression, confirming that Sema-3a-dependent repulsion does not require calcium signalling or Homer1 expression (Figure 3A). These data strongly suggest that reducing Homer1 expression may change the operational state of a CaMKII-CaN molecular switch, such that CaN-mediated repulsion is activated in response to signalling from BDNF.

If Homer1 knockdown does indeed cause BDNF to signal through CaN, it would be predicted that the inhibition of CaMKII signalling would have no effect on Homer1 morphant response to BDNF. Accordingly, inhibition of CaMKII with KN-93 had no effect on Homer1 morphant turning (Figure 3C). However, control morphant turning responses to BDNF were reversed from attraction to repulsion by KN-93 (Figure 3C). This confirms previous work demonstrating that attraction to BDNF is mediated by the CaMKII pathway [8]. KN-92, the inactive analog of KN-93, had no effect on control or Homer1 morphant turning in response to BDNF (Figure 3C). Inhibition of CaMKII by KN-93 did not change growth cone repulsion to Sema-3A gradients irrespective of Homer1 expression (Figure 3C). Generally, the pharmacological interventions outlined above did not affect overall axon extension (Figure 3B, D), confirming that inhibition of the CaMKII-CaN molecular switch did not perturb cytoskeletal rearrangements required for growth cone extension. Taken together, these data suggest that the activation state of the CaMKII-CaN molecular switch is contingent on appropriate levels of Homer1 expression in DRG growth cones.

### Homer1 is required for guidance cue activation of intracellular calcium stores

**Figure 4:**
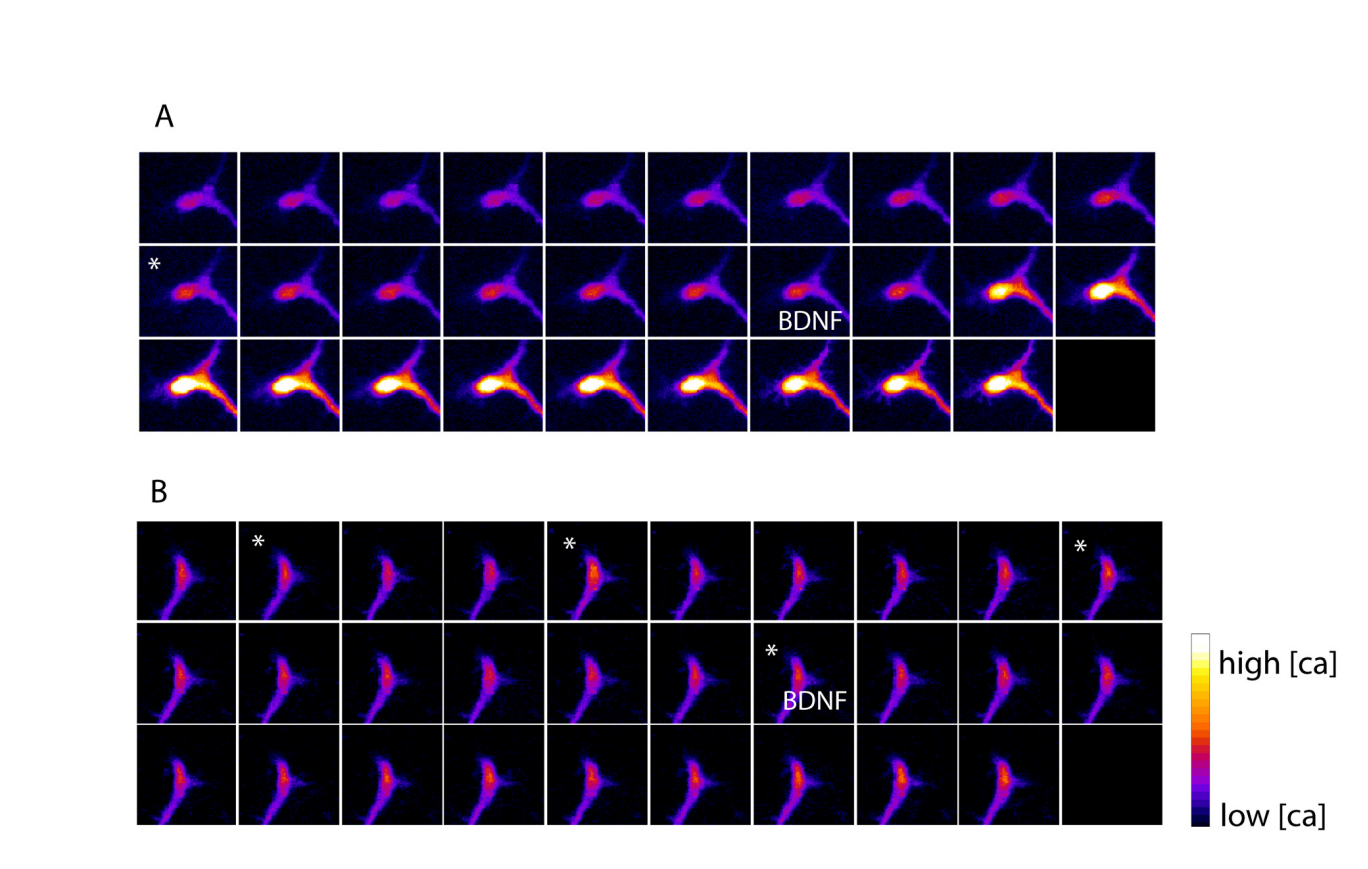
**Single wavelength calcium imaging of dorsal root ganglia growth cones in response to a microgradient of brain derived neurotrophic factor (BDNF)**. **(A) **Control morphant growth cone showing robust calcium flux in response to a BDNF gradient. (**B) **Homer1 morphant growth cone showing dramatic reduction of BDNF-induced calcium release. Frame interval is 18 s. The BDNF microgradient was established at the 'BDNF' frame. Frames marked with asterisks indicate spontaneous calcium transients.

**Figure 5:**
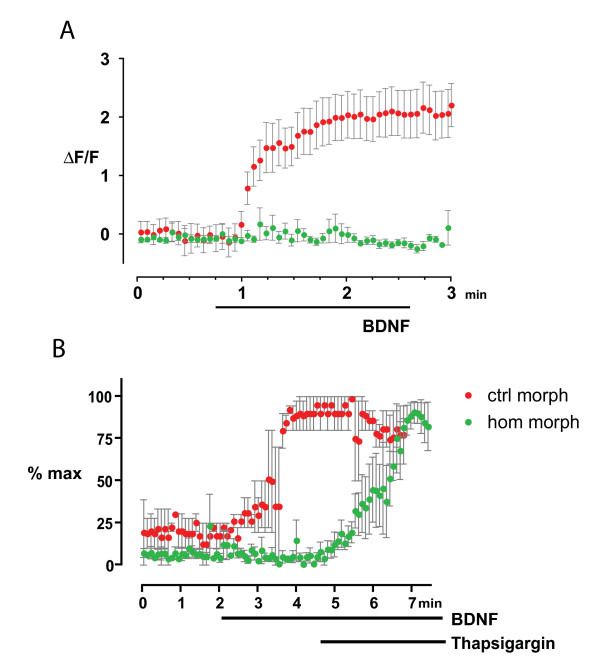
**Homer1 knockdown perturbs dorsal root ganglia growth cone calcium dynamics**. **(A) **Control (closed red circles) morphant growth cones (n = 9) exposed to a brain derived neurotrophic factor (BDNF) micro-gradient show significantly more calcium flux than Homer1 (closed green circles) morphant growth cones (n = 16). **(B) **Bath application of thapsigargin 2 minutes after establishment of a BDNF micro-gradient elicited a robust rise in intracellular calcium in Homer morphants (hom morph) but failed to elicit any rise in calcium in control morphants (ctrl morph). Error bars indicate standard error of the mean.

If Homer1 expression regulates the operational state of the CaMKII-CaN molecular switch, then it would be predicted that calcium dynamics within Homer1 morphant growth cones would be perturbed. We used single wavelength calcium imaging with the calcium indicator Fluo-4 to examine whether changes in Homer1 expression would alter calcium dynamics within turning growth cones. In control morphants there was a robust increase in calcium flux within 1 minute of exposure to BDNF that persisted as long as the gradient was present (Figures 4A and 5A), consistent with the findings of others [6,34,35]. In Homer1 morphants, the BDNF-induced rise in calcium flux was dramatically reduced (Figures 4B and 5A). Quantification of calcium flux during a BDNF gradient confirmed that treatment with the Homer1 morpholino virtually abolished the BDNF-induced rise in [Ca^++^]_i _seen in growth cones treated with the control morpholino (Figure 5A).

It is well established that BDNF-induced increases in [Ca^++^]_i _within growth cones requires the release of calcium from intracellular stores [1,3,6]. We then asked why the sustained rise in [Ca^++^]_i _in Homer1 morphant cells was absent: were intracellular stores defective or empty, or did Homer1 knockdown block signalling to IP_3_/ryanodine receptors to effect store release upon guidance cue activation? In order to determine if Homer1 morphants possessed functional calcium stores, we used acute bath application of thapsigargin (50 nM) in conjunction with exposure to a micro-gradient of BDNF. Acute application of thapsigargin mobilizes calcium by preventing re-uptake into intracellular stores [7,41,42]. In control morphants, thapsigargin did not elicit any further rise in [Ca^++^]_i_, suggesting that IP_3_-sensitive stores were depleted in response to BDNF stimulation (Figure 5B). Conversely, there was a robust thapsigargin-induced increase in [Ca^++^]_i _in Homer1 morphants (Figure 5B). These data demonstrate that the intracellular stores were not depleted in Homer1 morphants; rather, Homer1 is required to signal store release upon BDNF stimulation.

### Homer1 attenuates spontaneous Ca^++ ^influx

**Figure 6:**
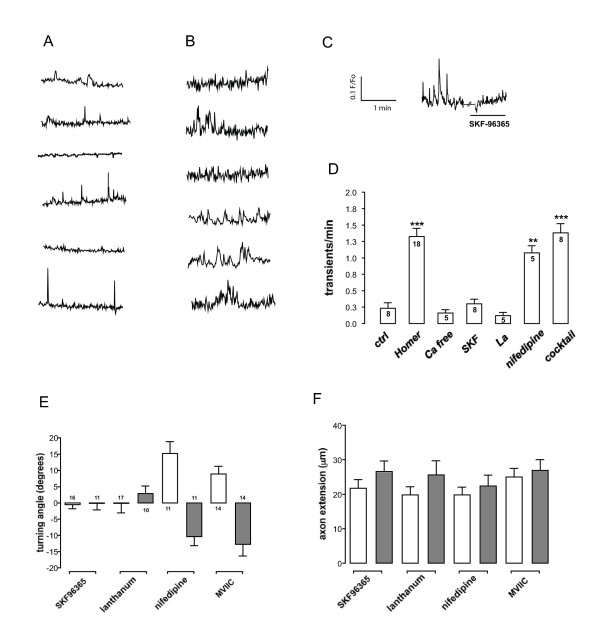
**Spontaneous calcium transients and growth cone turning are sensitive to blockage of store-operated channels**. **(A) **Individual control morphant growth cones exhibited sparse spontaneous calcium transients, occurring at a rate of approximately one transient per three minutes. **(B) **Homer1 morphant growth cones exhibited significantly greater frequency, at a rate of at least one spontaneous transient per minute. **(C) **A trace from a single Homer1 morphant growth cone showed a decrease in spontaneous calcium transient frequency in the presence of bath applied SKF-96365. **(D) **Quantification of spontaneous calcium transient frequencies in Homer1 morphant growth cones. Removing calcium from the media (Ca free) or bath application of La^3+ ^(La) or SKF-96365 (SKF) reduced spontaneous transient frequencies in Homer1 morphant growth cones to control (ctrl) levels. Bath application of a voltage-gated calcium channel (VGCC) inhibitor cocktail or nifedipine alone had little effect on the frequency of spontaneous calcium transients in Homer1 morphant growth cones. **(E) **Calcium-dependent brain derived neurotrophic factor (BDNF)-induced turning is mediated through store-operated channels. BDNF attraction was abolished when TRPC channels were inactivated with bath application of SKF-96365 or La^3+^. Inhibition of VGCCs with nifedipine or ω-conotoxin-MVIIC had no effect on control and Homer1 morphant growth cone turning. **(F) **Inhibition of store-operated channels did not alter axon extension rates. Error bars indicate standard error of the mean. Cocktail = nifedipine, ω-conotoxin-MVIIC plus Ni^++^. The scale bar in (C) applies also to (A, B).

The effect of Homer1 knockdown on guidance-cue-induced calcium signalling was profound (Figures 4 and 5). A more detailed analysis of calcium signals prior to the establishment of a BDNF gradient revealed a second change to calcium dynamics: a significant increase in the frequency of spontaneous calcium transients in Homer1 morphants (Figure 4B, asterisks; Figure 6B, D) compared to control morphants (Figure 6A, D). The frequency of spontaneous transient events in control morphants was comparable to that described previously [7]. In Homer1 morphants spontaneous transients were significantly attenuated in calcium-free media containing EGTA (300 μM; Figure 6D), confirming the extracellular source of calcium influx. The integral of such an increase in spontaneous calcium transients is likely to be reflected as an increase in basal cytosolic calcium [7] and these results thus suggest a crucial function for Homer1 in the maintenance of basal cytosolic Ca^++ ^within motile growth cones.

The demonstrated ability of Homer proteins to bind and gate TRPC channels [20] could account for the observed changes in BDNF-induced and spontaneous calcium dynamics. While TRPC channels may be activated by many stimuli, they are known to also function as store-operated channels, highly sensitive to intracellular calcium store depletion [26,43]. The frequency of spontaneous events observed in Homer1 morphants was significantly attenuated to control morphant levels by inhibitors of store-operated channels, SKF-96365 (3 μM) and lanthanum (100 μM La^3+^) (Figure 6C, D) [44,45] to control morphant levels (Figure 6D). While these compounds are used extensively to block TRPC channels, the spontaneous transients could also have arisen from calcium influx via voltage-gated calcium channels (VGCCs). We used the VGCC inhibitor nifedipine (5 μM) to target L-type VGCCs, or a cocktail containing nifedipine (5 μM), ω-conotoxin MVIIC (1 μM; to block N-, P- and Q-type VGCCs), and nickel (Ni^++^; 50 μM; to block T-type VGCCs). These VGCC inhibitors failed to significantly reduce the frequency of spontaneous calcium transients (Figure 6D). These data strongly suggest that the spontaneous Ca^++ ^transients were derived from influx through store-operated channels, potentially TRPC channels.

We used bath application of store-operated channel inhibitors and VGCC inhibitors in the turning assay to determine the contribution of these channels to Homer1 morphant growth cone turning. We confirmed that control morphant turning towards BDNF was abolished with bath application of SKF-96365 and La^3+^, consistent with the data of others [34,35]. Similarly, Homer1 morphant responses to BDNF were also abolished (Figure 6E), suggesting that growth cone turning relies on calcium influx through store-operated channels, possibly TRPC or Orai1 channels [30]. The VGCC inhibitors nifedipine, ω-conotoxin MVIIC and Ni^++ ^(data not shown) were used in an attempt to reveal any VGCC modulation of Homer1 morphant turning. However, there was no significant effect of VGCC inhibitors on control- or Homer1 morphant turning. Taken together, our data strongly suggest that Homer1 interacts with store-operated channels to regulate growth cone responses to calcium-dependent guidance cues.

### Close association of Homer1 and store-operated channels in growth cones

**Figure 7:**
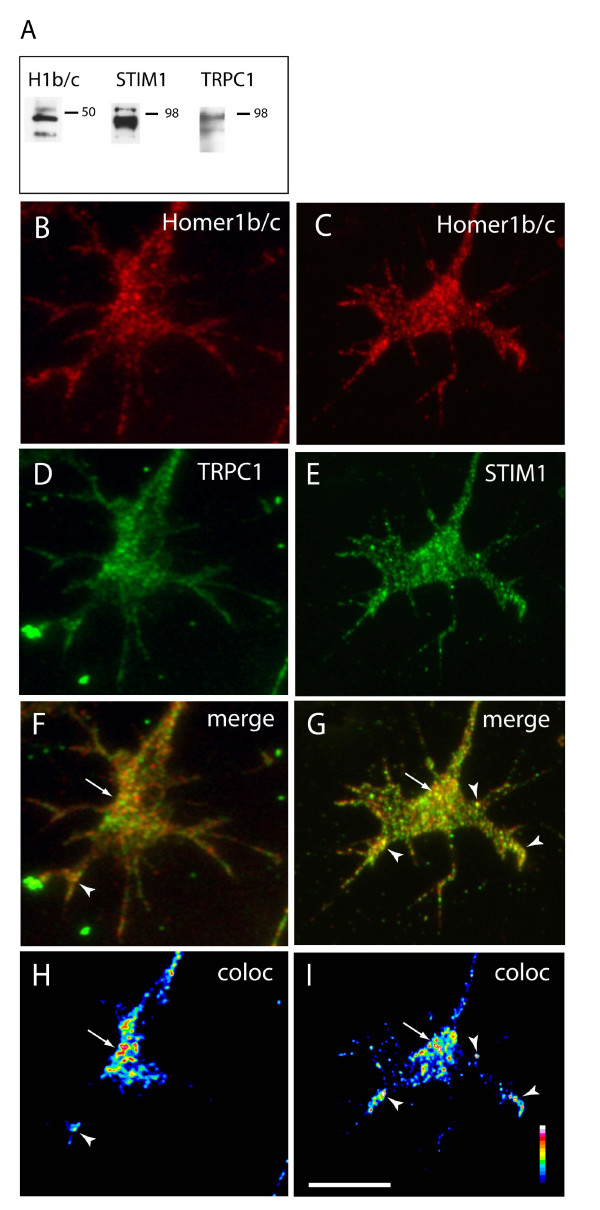
**Dorsal root ganglia (DRG) express Homer 1b/c, TRPC1 and STIM1 proteins in functionally relevant areas of growth cones in a pattern of close apposition and/or co-localisation**. **(A) **Western blot analysis of DRG tissue probed with Homer1b/c (H1b/c), TRPC1 and STIM1 antibodies. **(B, D) **Immunostaining of DRG growth cones revealed a punctate expression pattern for Homer1b/c (B, red) and TRPC1 (D, green). **(C, E) **Immunostaining revealed a punctate expression pattern for Homer1b/c (C, red) and STIM1 (E, green) in DRG growth cones. **(F, G) **Merged red/green images show close association of Homer1b/c with TRPC1 (F) and STIM1 (G). **(H, I) **Analysis of colocalisation probability depicted by pseudo-coloured images (white = high probability; blue = low probability) underscores the close apposition of Homer1b/c, TRPC1 and STIM1 proteins in functionally relevant areas of growth cones such as central area (arrows) and distal filipodial tips (arrowheads). Scale bar: 5 μm.

In order for Homer1 to gate store-operated channels and regulate [Ca^++^]_i _in motile growth cones, it would be predicted that Homer1 protein and store-operated channels are co-localised in functionally relevant structures, such as filopodia. We used immunofluorescence to compare growth cone localisation of Homer1b/c and the known store-operated proteins TRPC1 and STIM1, the ER calcium sensor for the store-operated channel Orai1. While there are extensive data in the literature regarding TRPC channels in DRG, Homer1 and STIM1 expression in DRG have not been reported previously. Western analysis demonstrated the presence of Homer1, TRPC1 and STIM1 proteins in DRG tissue (Figure 7A). Homer1b/c was constitutively expressed in DRG growth cones (Figure 7B, C), consistent with previous work showing Homer1b/c expression in developing sensory nervous systems [13,46]. Homer1b/c, TRPC1 and STIM1 all displayed a punctate pattern of immunoreactivity, prominent in the central zone and along filopodia (Figure 7B–E; TRPC3 and TRPC6 showed similar expression patterns (data not shown)). Image analysis revealed a close association or potential co-localisation of Homer1b/c with TRPC1 and STIM1 in functionally relevant areas of the growth cone such as the central area, distal filopodial tips and along filopodial shafts (Figure 7F–I). ER is known to extend into growth cone filopodia [47], so it was not surprising to observe the ER protein STIM1 in the DRG growth cone periphery. These localisation data support the notion that Homer1 regulates calcium influx via store-operated channels in growth cone turning.

## Discussion

This is the first study to examine the function of endogenous Homer1 protein in axon guidance. Knockdown of Homer1 expression reversed growth cone turning responses to calcium-dependent guidance cues from attraction to repulsion. Subsequently, Homer1 knockdown was shown to change the operational state of the CaMKII-CaN molecular switch. In keeping with a proposed crucial role in calcium signalling, Homer1 knockdown caused significant disturbances in calcium dynamics, abolishing the BDNF-induced release of stored calcium and causing an increased frequency of spontaneous calcium transients. Homer1-knockdown-induced growth cone turning and calcium transients were sensitive to inhibitors of store-operated channels. The close immuno-localisation of Homer1 with the store-operated proteins TRPC1 and STIM1 provided further evidence that Homer1 functions to mediate the activity of store-operated channels, thereby regulating intracellular calcium signalling and growth cone turning.

### Homer1 interacts with the CaMKII-CaN molecular switch

Homer1 knockdown did not simply change growth cone turning from attraction to random growth, but consistently reversed turning from attraction to repulsion in response to the calcium-dependent guidance cues Netrin-1 and BDNF. This reversal is significant in that it suggests that Homer1 modulates a proposed molecular switch that controls growth cone turning in response to calcium-dependent guidance cues. Currently, we know of a molecular switch in growth cones that is mediated by CaMKII-CaN and the relative levels of the cyclic nucleotides cAMP and cGMP [8,48-50]. Increased levels of cAMP effect attraction while increased cGMP levels repulsion and it is thought that the ratio of cAMP to cGMP likely modulates the turning switch *in vivo *[50,51]. The operational dynamics of the CaMKII-CaN molecular switch are sensitive to both baseline [Ca^++^]_i _and the depth of calcium signalling gradients, whereby a large change in [Ca^++^]_i _activates CaMKII and subsequent attraction. Alternatively, a small change in [Ca^++^]_i _activates CaN and subsequent repulsion [8]. The cyclic nucleotides work in concert with the CaMKII-CaN switch; for example, increased levels of cAMP, induced by high calcium, activate protein kinase A, which represses CaN, thereby facilitating growth cone attraction [8]. Our pharmacological experiments extend this model and demonstrate that attractive turning mediated by CaMKII requires Homer1 expression. Lowering Homer1 expression switched growth cone turning to the CaN repulsive pathway. This requirement of Homer1 in setting the CaMKII-CaN activation state suggested that Homer1 functions in regulating calcium dynamics within the growth cone. Calcium imaging experiments confirmed that Homer1 knockdown profoundly altered cytosolic calcium dynamics on two levels: a BDNF-induced rise in cytosolic calcium observed in control morphants was almost abolished in Homer1 morphants; and there was a significant increase in the frequency of spontaneous calcium transients in Homer1 morphants. Both results are consistent with Homer1 setting the operational state of the CaMKII-CaN switch: low Homer1 expression precludes a release of calcium from intracellular stores, and increases spontaneous transients, thereby perturbing basal cytosolic calcium and culminating in a shallow calcium signalling gradient.

An obvious mechanism by which Homer1 could have such a profound effect on calcium signalling is through its documented interactions with key calcium regulatory proteins, TRPC channels, IP_3 _and ryanodine receptors [16,20]. We propose a model whereby Homer couples with TRPC channels, IP_3_R and STIM1 into at least two signalling complexes: TRPC-Homer1-IP_3_R and TRPC-Homer-STIM1. The formation of these complexes relies on functional EVH1 and coiled-coil domains, consistent with previous work demonstrating the requirement for Homer1 in axon guidance *in vivo *[14]. That study demonstrated the absolute requirement of the coiled-coil and EVH1 domains of long form Homer1, although the exact mechanism of Homer function was not determined [14]. Homer1 knockdown would be predicted to disassemble these signalling complexes and disruption of a TRPC-Homer1-IP_3_R/ryanodine receptor complex would be predicted to have two effects: interference with calcium release via IP_3_R/ryanodine receptor gated intracellular calcium stores, consistent with the observed lack of a BDNF-induced rise in intracellular calcium; and to cause TRPC channels in the plasma membrane, which are normally activated by receptor activation and/or store depletion, to be spontaneously active [20,43], consistent with the observed increase in spontaneous calcium transients. Disruption of a putative TRPC-Homer1-STIM1 complex would be predicted to disrupt store-operated calcium entry [26], causing a loss of the sustained rise in intracellular calcium induced by extracellular guidance cues. These protein interactions would be consistent with Homer1 interacting with the CaMKII-CaN molecular switch within growth cones.

### Homer1 modulation of the molecular switch likely depends on interactions between Homer1 and store-operated proteins

Our turning and immuno-localisation data suggest a functional interaction between Homer and store-operated proteins in growth cone motility. Proteins known to regulate store-operated calcium entry include the TRPC family of cation channels and the Orai-STIM1 complex [30]. The close association of Homer, TRPC1 and STIM1 in key signalling regions of the growth cone suggest that STIM1-Homer-TRPC or IP_3_R-Homer1-TRPC coupling is well placed to transduce signals from extracellular cues and initiate filopodial calcium transients, which are crucial to the spatial and temporal regulation of calcium signalling in growth cones [5,52]. There are limited reports of STIM1 and Orai expression in neurons [27,53,54] and this is the first report demonstrating STIM1 expression in growth cones. However, recent work demonstrating the requirement for STIM-Orai signalling in the development of flight in *Drosophila *highlights the importance of STIM signalling in neuronal development [55]. There is a substantial body of evidence implicating TRPC channel function in neurons and, specifically, their requirement for growth cone turning. Calcium dependent turning of *Xenopus *spinal neurons requires xTRPC1 signalling [35,56]; similarly, hippocampal neurites require signalling through TRPC5 [57]. In cerebellar granule cells, the calcium-dependent guidance cues BDNF and netrin-1 signal through TRPC3 and TRPC6 channels [34]. These studies concluded that BDNF and Netrin-1, through their respective receptors TrkB and DCC, either directly or indirectly activate TRPC channels [34,35]. Similarly, we found that BDNF-induced turning and spontaneous calcium transients in Homer1 morphants were sensitive to inhibition of store-operated channels. Conversely, inhibition of voltage-dependent calcium channels had little effect on turning or spontaneous calcium transients. Notably, SKF-96365 and La^3+ ^blocked control and Homer1 morphant turning, confirming that store-operated channels act upstream of Homer1. While TRPC3, TRPC5 and TRPC6 have been implicated in growth cone motility [34,57], SKF-96365 and La^3+ ^are not selective for TRPC channels and block many TRP channels. In addition, TRPC channels form STIM1-mediated hetero-multimers in the plasma membrane [26]. Homer1 has been shown to interact directly with TRPC1, 2 and 5 [20]. It is likely, therefore, that TRPC homo- and hetero-tetramers [58] combine with IP_3_R or STIM1 and Homer1 tetramers to form dynamic signalling hubs [18] within growth cones.

The requirement of Homer1 in intracellular calcium store release was confirmed using acute application of thapsigargin. Thapsigargin inhibits smooth ER calcium/ATPase pumps, thus preventing uptake of calcium into stores [42]. Acute application of thapsigargin manifests itself as a rapid increase in [Ca^++^]_i _[7,42]. In control morphants, a robust rise in [Ca^++^]_i _was observed in response to BDNF. There was little or no additional calcium release after thapsigargin addition in control morphants, confirming efficient release of store calcium in response to BDNF. The subsequent sustained [Ca^++^]_i _rise in response to BDNF is likely due to store-operated calcium entry after store depletion [36,59,60]. Conversely, Homer1 morphants showed little or no increase in [Ca^++^]_i _in response to BDNF; however, a robust increase in [Ca^++^]_i _could be elicited by thapsigargin. There are at least two interpretations of these data: first, Homer1 knockdown does not interfere with filling of internal stores and, hence, Homer1 does not interact with smooth ER calcium/ATPase pumps; and second, Homer1 is required to trigger store release upon BDNF signalling through TrkB receptors and subsequent TRPC activation. This result is consistent with the role of Homer1 acting to couple TRPC and IP_3_Rs (or ryanodine receptors), thus gating TRPC activity as previously described in non-neuronal cells [20] and, thereby, regulating store-operated calcium entry in growth cones.

## Conclusion

Understanding the complete molecular repertoire that regulates calcium signalling in growth cones is a fundamental step in understanding the wiring of the nervous system. The data presented here support the hypothesis that the post-synaptic scaffolding protein Homer1 also acts pre-synaptically to mediate the activity of store-operated channels in growth cones to regulate crucial aspects of calcium signalling in response to guidance cue receptor activation. The exact nature and identity of the store-operated channels remains to be determined, although TRP channels are known as the 'sensory apparatus' of the cell [43] and this is particularly true in DRG, where they mediate a variety of sensory modalities such as temperature [61] and nociception [62]. Conversely, little is known of the intracellular calcium sensing proteins STIM1 and STIM2 in neurons. Given the extreme sensitivity of growth cones to external guidance cues [63] and their reliance on intracellular calcium signalling for responses to the external environment, it would seem likely that STIM proteins are important for correct growth cone navigation. Indeed, extracellular calcium sensing molecules have recently been shown to be important in neurite outgrowth and branching [64]. Homer is perfectly poised to integrate the function of STIM and TRPC proteins, and hence orchestrate the activity of store-operated proteins in the growth cones. Such a role would explain the gross axon guidance errors and target recognition failures observed *in vivo *after Homer1 mis-expression [14], since it would indicate that Homer1 signalling is required to mediate crucial aspects of calcium signalling, including CICR, store-operated calcium entry and maintenance of basal cytosolic calcium.

## Materials and methods

### Cell culture

Thoracic DRG from day 16 to 18 Hooded Wistar rat embryos were mechanically dissociated into sensory neuron medium (SNM) comprising Dulbecco's Modified Eagle's Medium/Ham's F-12 medium 1:1, (Gibco Biosciences, Carlsbad, CA, USA), fetal calf serum (5% v/v), penicillin G (100 U/ml), streptomycin (100 μg/ml), nerve growth factor (50 ng/ml; Sigma-Aldrich, St Louis, MO, USA) and N2 neural medium supplement (Gibco, Carlsbad, CA, USA). Morpholinos were loaded into neurons with a modification of a previously published method [65]. Briefly, whole DRG were vigorously and repetitively triturated through a 200 ml pipette tip in the presence of either fluorescein or biotin-labelled morpholino oligonucleotides (5 μM in SNM). Subsequent incorporation of morpholinos into neuronal cytosol was confirmed by either fluorescence or anti-biotin immunocytochemistry (data not shown). Cells were plated at low density onto poly-ornithine-(1 mg/ml; Sigma) and Laminin-(50 ng/ml; Gibco, Carlsbad, CA, USA) coated glass coverslips embedded into 35 mm plastic dishes (Iwaki, Asahi, Tokyo Japan).

### *In vitro *growth cone turning assay

Turning assays were performed as previously described [34,35,38]. Briefly, a molecular gradient was generated by the pulsatile ejection of the guidance cues BDNF (10 μg/ml), Netrin-1 (5 μg/ml) and Sema-3a (20 μg/ml) from fire-polished, modified patch micropipettes (tip diameter 1.0 to 1.2 μm). Concentrations of guidance cues at the growth cone were estimated as being 10^-3 ^of that in the pipette [38]. Isolated growth cones were imaged in SNM using phase contrast time-lapse microscopy. Multiple images were acquired and averaged every 7 s for 30 minutes using custom acquisition and data analysis software (MatLab, MathWorks, Natick, MA, USA). Turning angles and axon extensions were measured using ImageJ (NIH, Bethesda, MD, USA). Only growth cones extending at least 10 μm in 30 minutes were used for analysis. Turning angles were defined as the change in axon trajectory of the distal 10 μm of axons compared to their initial trajectories. Attraction and repulsion were designated positive and negative angles, respectively. Micropipette tips were positioned 80 to 100 μm from growth cones at a 45 degree angle to axonal trajectories. Unless otherwise stated, pharmacological agents were added to SNM 20 minutes prior to commencement of imaging and remained in culture medium for the duration of the turning assay. Statistical analysis of turning angles (Mann-Whitney *U*-test) were performed using Prism 4 (GraphPad Software, La Jolla, CA, USA).

### Immunofluorescence

Embryonic rat DRG cultures were fixed in 4% paraformaldehyde at room temperature for 4 h followed by permeabilisation and blocking with 0.4% Triton X-100 and 10% goat serum. Primary antibodies against Homer1b/c (1:100 to 1:500; Santa Cruz Biotechnology, Santa Cruz, CA, USA), TRPC1, TRPC3 and TRPC6 (1:100; Alomone Labs Jerusalem, Israel) and STIM1 (1:100; Sigma-Aldrich, St Louis, MO, USA) were added to coverslips overnight at 4°C. Controls for immunolabelling were performed by omitting the primary antibody in each case (data not shown). Detection of primary antibodies was performed using fluorescently labelled goat anti-mouse or goat anti-rabbit antibodies (Molecular Probes, Eugene, OR, USA). Actin labelling was with Phalloidin-Alexa 488 (5 U/ml; Molecular Probes, Eugene, OR, USA) added to the coverslips for 20 minutes prior to mounting. Homer1 knockdown images were acquired on an Olympus BX50 microscope equipped with a 50× oil immersion lens (NA 1.3). Homer1b/c and TRPC1 images were acquired with an Olympus IX80 inverted microscope (50× oil immersion lens, NA 1.3) equipped with a DSU spinning-disk confocal option. Homer1b/c and STIM1 images were acquired with a Zeiss LSM 510 laser confocal imaging system. Owing to the thinness of DRG growth cones *in vitro *(<1 to 2 μm), at least 2 to 3 Homer 1b/c, TRPC1 and STIM1 optical sections were acquired at 0.8 μm resolution and subsequently merged. When quantifying knockdown of Homer1 protein in growth cones, care was taken to eliminate any bias. Growth cones were selected while viewing actin staining and selected based on spreading morphology and isolation from other growth cones. In addition, exposures were set at a level so as to prevent pixel saturation and remained constant for all control and Homer1 morphant growth cones. Images were processed using ImageJ (NIH, Bethesda, MD, USA), Adobe Photoshop CS and Adobe Illustrator (Adobe Systems, San Jose, CA, USA). Co-localisation was analysed using the Colocalisation Test plugin (Image J, NIH, Bethesda, MD, USA [66]).

### Immunoblotting

For quantification of Homer1 knockdown, cells from a neuroblastoma cell line (B-35; American Type Culture Collection (ATCC), Manassas, VA, USA) were loaded with control and Homer1 morpholinos (5 μM) in a manner identical to that followed for DRG. Following incubation for 12 or 24 h, cells were harvested then lysed into RIPA buffer (50 mM Tris pH 7.4, 150 mM NaCl, 1 mM phenylmethylsulphonyl fluoride, 1 mM EDTA, 5 μg/ml aprotinin, 5 μg/ml leupeptin, 1% Triton X-100, 1% Na deoxycholate, 0.1% SDS). Total protein (30 μg) was separated on 12% SDS-PAGE, electroblotted onto 0.2 μm PVDF membranes, then blocked overnight in blocking solution (Boehringer-Mannheim, Mannheim, Germany). Membranes were incubated overnight at 4°C in primary antibody against Homer1b/c (1:1,000; a generous gift of Paul Worley, Johns Hopkins, MD, USA), rinsed thoroughly, then detected with goat anti-rabbit-HRP secondary antibodies for 2 to 3 h at room temperature. Conjugates were detected using ECL chemiluminescence reagent (Pierce, Rockford, IL, USA). Membranes were stripped (Restore PLUS, Pierce, Rockford, IL, USA) and re-probed with GAPDH primary antibody (1:1,000; Sigma-Aldrich, St Louis, MO, USA), secondary antibody and detected using ECL chemiluminescence reagent (Pierce, Rockford, IL, USA) to control for protein loading. Expression of Homer1, TRPC1 and STIM1 in whole DRG was confirmed using the same immunoblotting procedure. The primary antibodies were against Homer1b/c (1:200; Santa Cruz) and TRPC1 (1:200; Alomone Labs, Jerusalem, Israel), STIM1 (1:1,000; Sigma-Aldrich); secondary antibodies were goat anti-mouse-HRP or goat anti-rabbit-HRP (Dako, Glostrup, Denmark).

### Calcium imaging

Four to six hours after plating, DRG were loaded with Fluo-4AM calcium indicator (1 μM; Molecular Probes) in artificial cerebrospinal fluid (aCSF; 137 mM NaCl, 5 mM KCl, 5.6 mM glucose, 20 mM HEPES, 0.6 mM KH_2_PO_4_, 0.5 mM Na_2_HPO_4_, 1.4 mM Ca, 0.9 mM Mg) for 7 minutes at 37°C, washed with fresh aCSF and incubated at 37°C for a further 15 to 20 minutes prior to imaging. Orientation of micro-pipettes and establishment of guidance cue microgradients were identical as in the turning assay. Images (5 to 25 ms exposure) were captured every 3 s using a cooled CCD camera (ORCA, Hamamatsu, Hamamatsu City, Shizuoka Pref. Japan) and fluorescence intensities were analysed using custom software (Matlab, Mathworks, Natick MA, USA). Spontaneous event frequencies in isolated DRG growth cones were evaluated using a modified Daubechies 4 discrete wavelet transformation and analysis algorithm (MatLab, Mathworks, Natick MA, USA).

### Reagents

Control (TGgTGAAcATAcGTTGTTgCCCgAT) and specific Homer1 (TGCTGAAGATAGTTGTTCCCCCAT) morpholine oligonucleotides labelled with either fluoro-isothiocyanate or biotin (designed by GeneTools, LLC, Philomath, OR, USA); KN-93 and KN-92 (Calbiochem, San Diego, CA, USA)); nerve growth factor and cyclosporin A (Sigma-Aldrich, St Louis, MO, USA); Semaphorin-3A and Netrin-1 (R&D Systems, Minneapolis, MN, USA); BDNF, thapsigargin, nifedipine and ω-conotoxin-MVIIC (Alomone Labs); SKF-96365 (TOCRIS, Bristol, UK); La^3+ ^and Ni^++ ^(Sigma).

## Abbreviations

aCSF: artificial cerebrospinal fluid; BDNF: brain derived neurotrophic factor; [Ca^++^]_i_: cytosolic calcium; CaMKII: calcium-calmodulin dependent protein kinase II; CaN: calcineurin phosphatase; CICR: calcium-induced calcium release; DRG: dorsal root ganglia; ER: endoplasmic reticulum; EVH: enabled-VASP homology; IP_3_: inositol triphosphate; IP_3_R: inositol triphosphate receptor; Sema-3a: Semaphorin-3a; SNM: sensory neuron medium; STIM: stromal interacting molecule; TRPC: transient receptor potential canonical; VGCC: voltage-gated calcium channel.

## Competing interests

The authors declare that they have no competing interests.

## Authors' contributions

RG carried out most of the experimental work, and contributed to the design of the study, analysis and drafting of the manuscript. CM conducted some of the western blots for STIM and Homer1b/c. MJWT conducted some of the turning assays. DCL contributed to data interpretation and drafting of the manuscript. LF conceived the study, contributed to the design, coordination and analysis of the study, conducted some of the turning assays and drafted the manuscript. All authors read and approved the final manuscript.
